# Chemical Characterization of Volatile Compounds of *Lantana camara* L. and *L. radula* Sw. and Their Antifungal Activity

**DOI:** 10.3390/molecules171011447

**Published:** 2012-09-27

**Authors:** Juliana Lanna Passos, Luiz Claudio Almeida Barbosa, Antonio Jacinto Demuner, Elson Santiago Alvarenga, Cleiton Moreira da Silva, Robert Weingart Barreto

**Affiliations:** 1 Department of Chemistry, Federal University of Vicosa, Av. P. H. Rolfs, s/n, 36570-000, Vicosa, MG, Brazil; 2 Department of Phytopathology, Federal University of Vicosa, Av. P. H. Rolfs, s/n, 36570-000, Vicosa, MG, Brazil

**Keywords:** Verbenaceae, essential oils, *Corynespora cassiicola*, *Lantana camara*, *L. radula*

## Abstract

A comparative study of the chemical composition of essential oils of two very similar species of the Verbenaceae family (*Lantana camara* and *L. radula*) revealed that the main components of essential oil of *L. camara* were germacrene-D (19.8%) and *E*-caryophyllene (19.7%), while those of *L. radula* were *E*-caryophyllene (25.3%), phytol (29.2%) and *E*-nerolidol (19.0%). We have hypothesized that the observed differences could contribute to the differentiated reaction of the two species of *Lantana* to the attack of the phytopathogenic fungi * Corynespora cassiicola*. An experiment, involving *C. cassiicola* cultivation in culture media containing volatile oils of the two species demonstrated that the oils of *L. radula* were more fungistatic than the oils of *L. camara*, in accordance with the *in vivo* observations. It is likely that *E*-nerolidol and phytol, only found in the oil of *L. radula*, play a significant role in the effects of *L. radula* on *C. cassiicola*.

## 1. Introduction

Metabolites produced by plants are highly diverse, having distinct functions according to their structure and the places where they are secreted in the plant [1]. The Verbenaceae family includes numerous species that are rich in essential oils produced by secretor trichomes [2,3,4]. These essential oils can protect the plants against herbivore attacks and pathogens [5]. Many studies on the chemical composition of such essential oils have been published [6,7,8,9,10].

The Verbenaceae species *Lantana camara* L. is native to tropical and subtropical America and has been dispersed throughout the World as a popular ornamental plant, becoming one of the World’s worst weeds [11]. *Corynespora cassiicola* (Berk. & Curt.) is an anamorphic fungus, which causes foliar spots in more than 70 species of plants worldwide [12]. This is capable of causing a severe disease on *L. camara* and to provoke defoliation and debilitation of the attacked plants [13,14]. Conversely, the essential oils produced by *L. camara* may have defense properties against pathogens since *Lantana* species are known to produce essential oils with antimicrobial activity [15,16,17,18]. The effect of *Lantana* oils on *C. cassiicola* and related fungi has never been investigated. *Lantana camara* and *L. radula* are two closely related species, as reflected by their close morphological similarity [19]. However, when plants of the two species are maintained side by side, clear differences such as odor and foliar texture are easily noticed. The empirical observation made on differences of smell intensity suggested that the volatile substances produced by these two species might have a different composition leading to this comparative study.

The species *L. camara* presents secreting trichomes and idioblasts, both dispersed on the mesophyllum [19]. These structures are known to secrete lipidic substances [10], alkaloids, sesquiterpene lactones and flavonoids [20]. The chemical composition of essential oils produced by *L. camara* from various origins has been reported [6,9,15,21,22]. However, no report was found on the chemical composition of the essential oils produced by *L. radula*. As in a separate unpublished phytopathological study, inoculation of *L. radula* with *C. cassiicola* under controlled conditions resulted only in minor disease symptoms as compared to a significant impact on *L. camara*. Also, to the best of our knowledge, there is no published record of *C. cassiicola* as a pathogen of *L. radula*, contrary to *L. camara*. Based on such information, it was then conjectured that *L. radula* oil could be involved in the different response of this plant species to inoculation with *C. cassiicola* as compared with *L. camara.* This work represents an additional contribution to the studies on the composition of essential oils produced by aromatic and medicinal plants carried out by our research group [23,24,25,26,27,28,29,30,31,32,33,34,35,36] and the investigation of bioactive substances produced by microorganisms [31,32]. It includes information on the composition of essential oils produced by *L. camara* and *L. radula*, and their effect on mycelial growth in *C. cassiicola*.

## 2. Results and Discussion

### 2.1. Volatile Oils

It was found that both plant species produced a very small amount of oil, but *L. camara* produced nearly three times more (0.09 ± 0.005% w/w) than *L. radula* (0.03 ± 0.006% w/w). The moisture content for the leaves of *L. camara* was 79 ± 0.51% and for *L. radula* 67 ± 0.01%. Previous studies of *L. camara* reported oil content ranging from 0.06% w/w to 0.22% w/w [6,21]. The components found for the oils of *L. camara* (19 compounds) and *L. radula* (nine compounds) are presented in Table 1.

**Table 1:** Major components identified in the volatile oils of *L. camara* and *L. radula*.

Peak no.	Constituent ^a^	RI	*L. camara (%)*	*L. radula (%)*
1	α-Copaene	1374	1.1 ± 0.12	-
2	β-Elemene	1390	3.2 ± 0.51	-
3	Tetradecane	1399	1.0 ± 0.55	1.8 ± 0.26
4	***E*-Caryophyllene**	1418	**19.7 ± 3.32**	**25.3 ± 5.47**
5	β-Gurjunene	1427	1.2 ± 0.15	-
6	**α-Humulene**	1451	**9.3 ± 0.30**	1.2 ± 0.06
7	β- *E-*Farnesene	1458	1.3 ± 0.20	Tr
8	**Germacrene-D**	1479	**19.8 ± 1.40**	**17.6 ± 1.21**
9	**Bicyclogermacrene**	1493	**11.7** ± **0.67**	4.0 ± 0.10
10	α-Muurolene	1498	1.5 ± 0.15	-
11	Germacrene-A	1501	2.5 ± 0.36	-
12	Cubeol	1511	2.5 ± 1.14	-
13	δ-Cadinene	1520	2.4 ± 0.35	-
14	***E*-Nerolidol **	1562	-	**19.0** ± **3.56**
15	Germacrene-D-4-ol	1567	1.2 ± 0.30	-
16	Caryophyllene oxide	1579	-	1.7 ± 0.67
17	Davanone	1572	1.2 ± 0.58	-
18	Globulol	1581	0.7 ± 0.40	-
19	Humuladyenone *	1594	1.2 ± 0.36	-
20	Epi-α-Muurolol	1638	4.8 ± 0.32	-
21	**Phytol**	2092	4.0 ± 1.45	**29.2** ± **5.23**
	Identified (%)		86.3	99.8

Tr: component aspect < 0.1; * Identification with GC-MS; ^a^ Compounds listed in order of elution; Retention Index (RI): measurement relative to alkanes in a BD-5 column.

The empirical observation made previously of a clear smell difference between the two species of *Lantana* was confirmed by gas chromatography and mass spectrometry analyses that revealed different compositions for their volatile oils. The common constituents for the two species are: tetradecane, *E*-caryophyllene, α-humulene, β-*E*-farnesene, germacrene-D, bicyclogermacrene and phytol. The major components in the oil of *L. camara* are germacrene D (19.8%), *E*-caryophyllene (19.7%), bicyclogermacrene (11.7%) and α-humulene (9.3%). These compounds represent approximately 60.5% of the oil. The four major constituents of the oil of *L. radula* are *E*-caryophyllene (25.3%), germacrene-D (17.6%), *E*-nerolidol (19.0%) and phytol (29.2%), representing 91.1% of the oil. 

The greater chemical diversity of the volatile oils of *L. camara* as compared to *L. radula* could be connected with the presence of secretor idioblasts in *L. camara* [19] which are absent in *L. radula* but further studies are required to confirm this hypothesis. 

### 2.2. Bioassay

**Figure 1:**
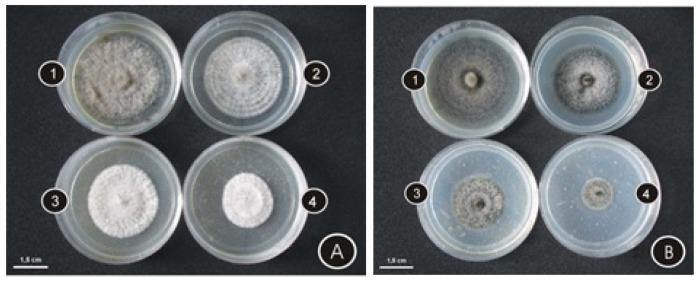
Colonies of *C. cassiicola* after eight days of growth in medium containing increasing amounts of oil obtained from *L. camara* (**A**) and *L. radula* (**B**) as compared to control: (1) control; (2) 3,000 mg L^−1^; (3) 5,000 mg L^−1^; (4) 10,000 mg L^−1^.

As observed on Figure 1, oils obtained from each of these species caused a significant inhibition on fungal growth, as compared to controls.

**Figure 2:**
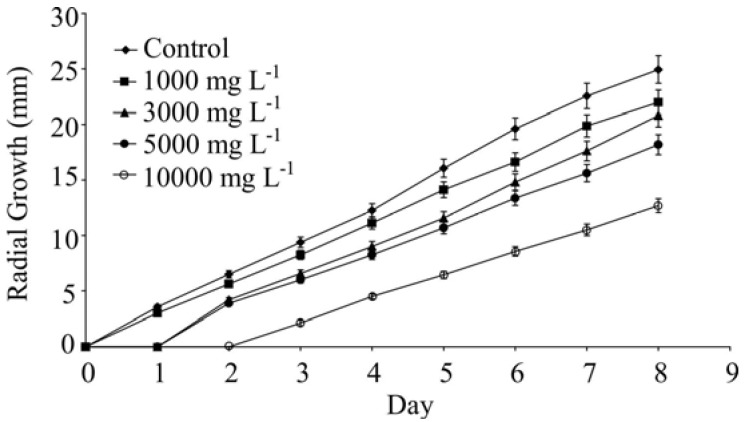
Radial culture growth of *C. cassiicola* in culture media containing *L. camara* volatile oils (LC) at concentrations of 1,000, 3,000, 5,000 and 10,000 mg L^−1^.

In the first two days of evaluation the oil of *L. camara* at a concentration of 1,000 mg L^−1^, caused a reduction in the growth of the fungal colonies of 25.0% and 19.6%, respectively. At higher concentrations the fungus growth was completely inhibited during the first day and for 10,000 mg L^−1^ a full inhibition was still observed during the second day (Figure 1A and Figure 2).

At the eighth day of evaluation the reduction of the development of the colonies was dependent on the oil concentration tested and the following results were found: 12% inhibition at 1,000 mg L^−1^; 17% at 3,000 mg L^−1^; 27% at 5,000 mg L^−1^ and 49% at 10,000 mg L^−1^. These results are similar to published results where inhibitory effect of the oil from *L. camara* on the growth of *Aspergillus niger* van Tiegh and *Fusarium solani* Mart. (Sacc) are reported [15].

**Figure 3:**
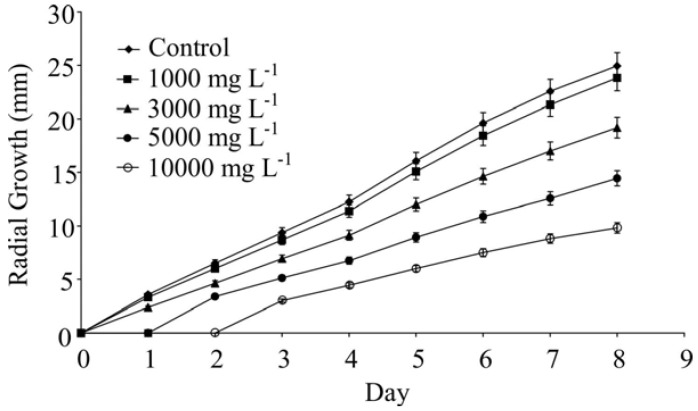
Radial culture growth of *C. cassiicola* in culture medium containing *L. radula* volatile oils (LR) at concentrations of 1,000, 3,000, 5,000 and 10,000 mg L^−1^.

For the oil of *L. radula* at the concentrations of 1,000 mg L^−1^ and 3,000 mg L^−1^, 17.2% and 40.6% inhibition were observed, respectively, on the first day of evaluation (Figure 1B and Figure 3). At higher concentrations (5,000 and 10,000 mg L^−1^) the oils caused complete inhibition of the fungus growth. Even after eight days of experiment the volatile oils of *L. radula* caused a significant reduction on fungus development (4.7% inhibition at 1,000 mg L^−1^; 23.2% at 3,000 mg L^−1^; 42.3% at 5,000 mg L^−1^ and 60.8% at 10,000 mg L^−1^).

The results demonstrated that the volatile oils from *L. radula* have a greater effect on the growth of *C. cassiicola* compared with the oils obtained from *L. camara.* Although bioassays with each individual component of oils from each plant species were not performed, it is likely that the greater activity of *L. radula*’s oil could be associated with its higher concentration of *E*-nerolidol, since this compound has already been shown highly active against *Escherichia coli* [37], to have larvicidal activity on *Aedes aegypti* larvae and anti-parasitic activity against a strain of *Leishmania amazonensis* [38]. Another significant difference between the oils is the higher concentration of phytol in *L. radula* oil, although there is no published report of any anti-fungal activity for this compound. Although caryophyllene is present in high concentration in both oils, previous investigation have demonstrated that it has no effect, at least against the basidiomycete species *Laetiporus sulphureus*, *Lenzites betulina* and *Trametes versicolor* [39]. The higher growth-inhibiting activity observed for *L. radula* oil on *C. cassiicola* may in part explain the observed resistance to attack by this fungus [19].

## 3. Experimental

### 3.1. Plant Material

Aerial parts of *L. camara* and *L. radula*, from plants grown in a greenhouse, were used. Voucher specimen of each plant species were deposited in the VIC Herbarium [registration numbers 30159 (*L. camara*) and 30160 (*L. radula*)] for reference.

### 3.2. Extraction and Analysis of the Essential Oils

Leaves of *L. camara* and *L. radula* were collected and each sample was subdivided into three portions of 40 g each and then subjected to 3 h of hydrodistillation in a Clevenger apparatus. The resulting oils were separated from the aqueous phase, weighed and the reported yields were calculated with respect to the mass of dry material. All distillations were repeated three times and the oils obtained were separated from the aqueous phase and stored under a nitrogen atmosphere, maintained at 0 °C, until they were chromatographically analyzed. Dry leaf weight was calculated by drying each sample (2 g, held at 103 ± 2 °C for 24 h) [40]. Each analysis was carried out in triplicate.

### 3.3. Essential Oil Gas Chromatography-Mass Spectrometry (GC-MS) Chemical Analysis

Qualitative analyses were conducted on a GC-MS-QP5050A system with a mass-selective detector (Shimadzu, Kyoto, Japan), equipped with a DB-5 (J & W Scientific, Albany, NY, US) fused silica column (30 m × 0.25 mm i.d., film thickness 0.25 µm). Column temperature was 40 °C (2 min), increased at 3 °C/min to 240 °C, and kept at this temperature for 10 min. Injector temperature was 220 °C. Helium was the carrier gas at a flow rate of 1.8 mL/min. An amount of 1 μL (1% w/v solution of the oil in dichloromethane) was injected and the split ratio was 1:10. The column pressure corresponded to 100 kPa. Mass detector conditions were as follows: temperature source 240 °C; electron impact (EI) mode at 70 eV; scan rate 1 scan/s; mass acquisition range 29–450 u.

The identification of the components was performed by comparison of their retention indexes (RI), relative to a standard alkane series (C_9_-C_24_), and comparison of the mass spectra with those on record in the Wiley library data base (Wiley 330000) or from the literature [41].

GC analyses were carried out in triplicate, and accomplished with a GC-17A series instrument (Shimadzu) equipped with a flame ionization detector (FID). Chromatographic conditions were as follows: Fused silica capillary column (30 m × 0.22 mm) with a DB-5 bonded phase (0.25 μm film thickness); carrier gas, N_2_ at a flow rate of 1.8 mL/min; injector temperature 220 °C, detector temperature 240 °C; column temperature was programmed to start at 55 °C (isothermal for 2 min), with an increase of 3 °C/min, to 240 °C, isothermal at 240 °C for 15 minutes; injection of 1.0 μL (1% w/v in dichloromethane); split ratio 1:10; column pressure of 115 kPa.

### 3.4. Effect of Volatile Oils on Mycelial Growth of C. Cassiicola

The oils obtained from each plant were dissolved in Tween 20 and tested *in vitro* for antifungal activity against *C. cassiicola*, using the “Poison Food Technique” [42]. Each oil was incorporated in the vegetable broth agar VBA [13] at 1,000, 3,000, 5,000 and 10,000 mg L^−1^ (Tween 0.1%), and were vigorously agitated and poured sterilized Petri dishes (60 mm in diameter). The plates were centrally seeded with culture disks (5 mm in diameter) obtained from the margin of actively growing cultures of *C. casiicola* f. ssp. *lantanae* (isolate JMP 17) from the culture collection of the Department of Plant Pathology at UFV and incubated at 25 ± 2 °C in the dark. Plates containing the fungus but without Lantana oil served as controls and were incubated under the same conditions. The incubation lasted 8 days in the dark. The effect of the oil on the mycelial growth (mm) was determined by measuring the radial growth of *C. cassiicola* in intervals from the 1st to the 8th day after the inoculation. The percentage of fungus growth inhibition was calculated in relation to the radial growth in control plates. Treatments were carried out in a completely randomized design with four replications and the data was analyzed by the Tukey’s test at 0.05 probability level.

## 4. Conclusions

The volatile oils obtained from the two species of *Lantana* had significantly different chemical composition and the oil produced by *L. camara* contained a greater diversity of compounds. Although chemical composition of *L. camara* oil has already been well investigated, no such data was found for *L. radula*. The greater effect of the volatile oil of *L. radula* compared with that produced by *L. camara* on the growth of *C. cassiicola* could in part explain its resistance to this fungal pathogen. The results of this study encourage further investigation on the effect of each of the major components of the oil from *L. camara* against plant pathogenic fungi, particularly against the dematiaceous species related to *C. cassiicola*.
